# Prevalence of infertility and help seeking among 15 000 women and men

**DOI:** 10.1093/humrep/dew123

**Published:** 2016-08-19

**Authors:** J. Datta, M.J. Palmer, C. Tanton, L.J. Gibson, K.G. Jones, W. Macdowall, A. Glasier, P. Sonnenberg, N. Field, C.H. Mercer, A.M. Johnson, K. Wellings

**Affiliations:** 1 Centre for Sexual and Reproductive Health Research, Department of Social and Environmental Health Research, London School of Hygiene & Tropical Medicine, 15-17 Tavistock Place, London WC1H 9SH, UK; 2 Research Department of Infection and Population Health, University College London, Mortimer Market Centre, London WC1E 6JB, UK; 3 MRC Centre for Reproductive Health, Queen's Medical Research Institute, 47 Little France Crescent, Edinburgh EH16 4TY, UK

**Keywords:** infertility, female infertility, male infertility, help seeking, population survey

## Abstract

**STUDY QUESTION:**

What is the prevalence of infertility and of help seeking among women and men in Britain?

**SUMMARY ANSWER:**

One in eight women and one in ten men aged 16–74 years had experienced infertility, defined by unsuccessfully attempting pregnancy for a year or longer, and little more than half of these people sought medical or professional help.

**WHAT IS KNOWN ALREADY:**

Estimates of infertility and help seeking in Britain vary widely and are not easily comparable because of different definitions and study populations.

**STUDY DESIGN, SIZE, DURATION:**

A cross-sectional population survey was conducted between 2010 and 2012 with a sample of 15 162 women and men aged 16–74 years.

**PARTICIPANTS/MATERIALS, SETTING, METHODS:**

Participants completed the Natsal-3 questionnaire, using computer-assisted personal interviewing (CAPI) and computer-assisted self-interview (CASI).

**MAIN RESULTS AND THE ROLE OF CHANCE:**

The reported prevalence of infertility was 12.5% (CI 95% 11.7–13.3) among women and 10.1% (CI 95% 9.2–11.1) among men. Increased prevalence was associated with later cohabitation with a partner, higher socio-economic status and, for those who had a child, becoming parents at older ages. The reported prevalence of help seeking was 57.3% (CI 95% 53.6–61.0) among women and 53.2% (CI 95% 48.1–58.1) among men. Help seekers were more likely to be better educated and in higher status occupations and, among those who had a child, to have become parents later in life.

**LIMITATIONS, REASONS FOR CAUTION:**

These data are cross-sectional so it is not possible to establish temporality or infer causality. Self-reported data may be subject to recall bias.

**WIDER IMPLICATIONS OF THE FINDINGS:**

The study provides estimates of infertility and help seeking in Britain and the results indicate that the prevalence of infertility is higher among those delaying parenthood. Those with higher educational qualifications and occupational status are more likely to consult with medical professionals for fertility problems than others and these inequalities in help seeking should be considered by clinical practice and public health.

**STUDY FUNDING/COMPETING INTEREST(S):**

Funding was provided by grants from the Medical Research Council and the Wellcome Trust, with support from the Economic and Social Research Council and the Department of Health. AMJ is a Governor of the Wellcome Trust. Other authors have no competing interests.

## Introduction

Fertility is a key element of reproductive health and infertility is recognized as a global public health issue by the World Health Organisation (WHO) (Boivin *et al*., 2007; Macaluso *et al*., 2010). Infertility is defined by the International Committee for Monitoring Assisted Reproductive Technology (ICMART) and WHO as the ‘failure to achieve a pregnancy after 12 months or more of regular unprotected sexual intercourse’ (Zegers-Hochschild *et al*., 2009). The National Institute for Health and Care Excellence (NICE) guideline for England and Wales concurs with this time period, advocating that clinical assessment and investigation should be offered when ‘a woman of reproductive age… has not conceived after one year of unprotected vaginal sexual intercourse, in the absence of any known cause of infertility’ (National Institute for Health and Clinical Excellence, 2013). The experience of infertility can cause those affected personal distress (Schneider and Forthofer, 2005; Greil *et al*., 2011), significant treatment costs (Bell, 2010) and, in some contexts, ostracism and discrimination (Cui, 2010).

Infertility is experienced by an estimated 48.5 million couples worldwide (Mascarenhas *et al*., 2012) and around 1 in 7 couples in the UK (National Institute for Health and Clinical Excellence, 2013). However, prevalence estimates of lifetime infertility vary widely, in part because there is no agreed or consistent definition of infertility (Gurunath *et al*., 2011) and because study populations vary by age range, unit of measurement and relationship status.

A marked trend for delaying the timing of first birth has been seen in developed and, more recently, developing countries (Gyimah, 2003; Mathews and Hamilton, 2009; Rosero-Bixby *et al*., 2009; Mills *et al*., 2011). A consequence of postponement of parenthood is the potential for impaired fertility (Balasch, 2010) and the risk that deferral of parenthood will rule it out (Sobotka, 2006; Leridon, 2008).

Later parenthood, its implications for fertility, and the increased range and availability of fertility treatments are likely to have had an impact on help seeking. There is some evidence of an increase in the reporting of fertility problems to primary care after the introduction of national treatment guidelines, suggesting more awareness of assisted reproductive technology (ART) (Dhalwani *et al*., 2013). Studies have found that women in later age cohorts are more likely to seek help than women in earlier ones and that women are seeking help at older ages than was previously the case (Oakley *et al*., 2008; Wilkes *et al*., 2009). There are few population-based data on experience of infertility and its determinants or on the extent of help seeking, particularly for men. This study uses data from the third National Survey of Sexual Attitudes and Lifestyles (Natsal-3) to estimate the prevalence of infertility, and of seeking medical or professional help among women and men in Britain, and to examine associated factors.

## Materials and Methods

### Study population

Natsal-3 is a survey of 15 162 women (8869) and men (6293) aged 16–74 years. Households were selected using stratified probability sampling from which one eligible individual resident in Britain was selected at random and invited to participate. The sample frame was the Postcode Address File (PAF), a regularly updated list of all addresses in the country. As the PAF excludes those who are homeless or living in institutions, our sample is representative of individuals living in private residential households. Data were weighted in two stages to correct for participants' unequal probabilities of selection. The first corrected for the selection of one household in multi-household addresses and for the varying probabilities of selection by number of adults within households. The second adjusted for differential non-response by comparing age, sex and region profile of participants with 2011 census data. Although the Natsal-3 sample closely matched those who responded to the census in terms of ethnicity (86.8% in Natsal-3 and 86.7% in the census were white), there was a slight under-representation of Asian women and men in Natsal-3 (6.4%) compared with the census (7.5%). Participants were interviewed between 2010 and 2012 using computer-assisted personal interviewing (CAPI), which included a computer-assisted self-interview (CASI) component for the more sensitive questions. The response rate was 57.7% and the co-operation rate (i.e. of all eligible addresses contacted) was 65.8%. Further details of the methods are described elsewhere (Erens *et al*., 2014).

Questions about experience of infertility and help seeking for infertility were asked of all Natsal-3 participants who reported ever having heterosexual intercourse (8315 women and 5742 men). Two discrete questions were asked: ‘*Have you ever had a time*, *lasting 12 months or longer*, *when you and a partner were trying for a pregnancy but it didn't happen?*’ and ‘*Have you (or a partner) ever sought medical or professional help about infertility?*’. In line with the NICE guideline (National Institute for Health and Clinical Excellence, 2013), we considered a participant to have experienced infertility when she or he responded ‘yes’ to the first question. Participants were also asked questions that comprise the validated Patient Health Questionnaire-2 (PHQ-2) (Kroenke *et al*., 2003), a composite measure of depression experienced in the 2 weeks prior to interview and questions on satisfaction with sex life and relationship in the past year.

Data are cross-sectional and we did not ask participants for information about when their experience of infertility occurred. Some may have failed to conceive in a 12-month period before becoming parents or between pregnancies while others may never have become parents. Those still of reproductive age could experience a future period of infertility. Natsal-3 data are deposited at the UK Data Service. https://discover.ukdataservice.ac.uk/catalogue/?sn=7799 (11 May 2016, date last accessed).

### Statistical analyses

We used STATA v13.1 (StataCorp, 2013) to undertake complex survey analyses to account for the weighting, clustering and stratification of the Natsal-3 data. We estimated the population prevalence of infertility and help seeking, stratified by gender and age group. We used multivariable logistic regression to explore associations (adjusted for age at interview) between experience of (i) infertility and (ii) help seeking, and a number of socio-demographic, relationship, reproductive and health factors, including: age at interview; relationship status at interview; age at first cohabitation; age at first child; academic attainment; employment status measured using National Statistics Socio-Economic Classification (NS-SEC) and area-level deprivation measured using the Index of Multiple Deprivation (IMD) (Office for National Statistics, 2010). We report results for educational attainment for those aged over 21 years only as younger participants may not have completed full-time education. Finally, using multivariable logistic regression, we present age-adjusted odds ratios to describe associations between experience of infertility and selected health and relationship ‘outcome’ variables for women aged 50 years or younger. We selected this age group for analysis as their experience of infertility will have occurred more recently than that of older participants.

### Ethical approval

The Natsal-3 study was approved by the Oxfordshire Research Ethics Committee A (ref.: 10/H0604/27).

## Results

### Prevalence of infertility and associated factors

The proportion of participants aged 16–74 who reported ever having tried unsuccessfully for a year or longer to become pregnant was 12.5% among women and 10.1% among men (Table I). Unsurprisingly, the prevalence of ever experience of infertility was lowest in the youngest women and peaked among those aged 35–44 years (17.7%). A similar pattern was seen for men but the age range in which the prevalence of ever experience of infertility was highest was wider than for women, extending from age 35 to 54 years at interview.

**Table I:** Prevalence of and factors associated with infertility by sex.

	Women						Men					
	Pregnancy attempt 12 months or longer		Age-adjusted regression				Pregnancy attempt 12 months or longer		Age-adjusted regression			
	%	95% CI	AOR	95% CI	*P*-Value	Denominators (unweighted, weighted)	%	95% CI	AOR	95% CI	*P*-Value	Denominators (unweighted, weighted)
All												
	**12.5%**	**(11.7–13.3)**				*8066*, *7052*	**10.1%**	**(9.2–11.1)**				*5553*, *6811*
Age at interview					<0.0001						<0.0001	
16–24	5.3	(4.3–6.6)	1.00			*1695*, *944*	3.7	(2.6–5.1)	1.00			*1325*, *971*
25–34	12.0	(10.7–13.5)	2.43	(1.87–3.17)		*2366*, *1306*	8.5	(7.0–10.2)	2.44	(1.62–3.67)		*1421*, *1274*
35–44	17.7	(15.7–20.0)	3.85	(2.92–5.06)		*1173*, *1403*	14.9	(12.4–17.8)	4.62	(3.10–6.87)		*780*, *1377*
45–54	12.5	(10.6–14.7)	2.56	(1.90–3.45)		*1062*, *1374*	14.5	(11.8–17.7)	4.48	(2.95–6.81)		*741*, *1328*
55–64	13.2	(11.0–15.6)	2.71	(2.00–3.66)		*972*, *1170*	7.8	(5.9–10.3)	2.24	(1.41–3.56)		*697*, *1094*
65–74	11.4	(9.2–14.0)	2.29	(1.65–3.18)		*798*, *854*	8.2	(6.1–11.0)	2.35	(1.47–3.74)		*589*, *767*
Relationship history												
Relationship status at interview					<0.0001						<0.0001	
Married/cohabiting	15.2	(14.1–16.4)	1.00	-		*4348*, *4669*	12.6	(11.3–14.0)	1.00	-		2925, 4640
Non-cohabiting partnership	6.1	(4.8–7.8)	0.39	(0.29–0.51)		*1355*, *786*	4.0	(2.7–5.8)	0.29	(0.18–0.45)		941, 755
No ‘steady’ partner	7.6	(6.5–9.0)	0.46	(0.38–0.57)		*2326*, *1573*	5.3	(4.2–6.7)	0.39	(0.29–0.51)		1653, 1384
Ever cohabited with a partner					<0.0001						<0.0001	
Never	2.2	(1.5–3.2)	1.00			*1499*, *855*	2.1	(1.3, 3.3)	1.00			*1481*, *1151*
Ever	13.8	(12.9–14.8)	7.50	(5.01–11.25)		*6350*, *6013*	11.8	(10.7, 13.0)	6.91	(4.13–11.55)		*3920*, *5465*
Age at first cohabitation					0.0685						0.8975	
Under 20	12.9	(11.3–14.7)	1.00			*1970*, *1685*	11.6	(9.0–14.9)	1.00			*574*, *677*
20–29	13.8	(12.7–15.1)	1.09	(0.91–1.30)		*3972*, *3856*	11.9	(10.6–13.4)	1.05	(0.77–1.44)		*2852*, *3982*
30 or older	17.6	(13.9–22.0)	1.45	(1.06–1.99)		*402*, *464*	11.1	(8.5–14.5)	0.98	0.65–1.48)		*465*, *763*
Reproductive history												
Ever had a child					0.0880						0.0070	
Yes	13.2	(12.2–14.3)	1.00			*5248*, *5110*	11.7	(10.4–13.2)	1.00			*2694*, *4031*
No	10.5	(9.2–11.9)	0.83	(0.68–1.03)		*2808*, *1935*	7.9	(6.7–9.3)	0.68	(0.52–0.90)		*2857*, *2775*
Age at birth of first child^b^					<0.0001						<0.0001	
<25	9.0	(7.9–10.3)	1.00			*3047*, *2709*	7.6	(5.9–9.7)	1.00			*946*, *1234*
25–29	13.2	(11.3–15.3)	1.54	(1.23–1.92)		*1337*, *1392*	10.2	(8.1–12.7)	1.44	(1.00–2.06)		*902*, *1363*
30–34	20.9	(17.6–24.6)	2.63	(2.03–3.40)		*616*, *709*	14.5	(11.4–18.3)	2.08	(1.41–3.08)		*520*, *852*
35+	35.1	(28.6–42.1)	5.57	(3.97–7.83)		*227*, *278*	19.8	(15.3–25.4)	3.27	(2.16–4.96)		*301*, *550*
Abortion ever					0.5855							
No	12.4	(11.5–13.3)	1.00	-		*6853*, *6046*						
Yes	12.9	(10.7–15.5)	1.07	(0.84–1.35)		*1176*, *968*						
Socio-economic position												
Educational level^a^					0.0006						0.5684	
Degree	14.5	(12.8–16.3)	1.00			*1938*, *1755*	12.0	(10.1–14.2)	1.00			*1375*, *1904*
A-level/equivalent	13.5	(11.6–15.7)	0.90	(0.72–1.14)		*1546*, *1420*	11.0	(9.1–13.1)	0.90	(0.68–1.19)		*1336*, *1762*
GCSE, O-level or equivalent	13.9	(12.4–15.6)	0.93	(0.76–1.13)		*2496*, *2294*	10.3	(8.6–12.4)	0.84	(0.64–1.12)		*1382*, *1750*
Foreign or other	11.3	(5.8–21.0)	0.76	(0.36, 1.62)		*86*, *67*	12.9	(5.8–26.5)	1.11	(0.44–2.77)		*46*, *57*
None	8.8	(7.1–10.9)	0.53	(0.40, 0.71)		*966*, *950*	9.2	(6.7–12.4)	0.73	(0.48–1.09)		*530*, *701*
Social class (NS-SEC)					<0.0001						0.0003	
Managerial and prof occupations	15.2	(13.5–17.0)	1.00			*2341*, *2230*	12.8	(11.1–14.6)	1.00			*1786*, *2514*
Intermediate occupations	13.5	(11.6–15.7)	0.87	(0.70, 1.09)		*1586*, *1426*	10.0	(7.9–12.6)	0.76	(0.55–1.03)		*871*, *1159*
Semi-routine/routine occupations	11.1	(9.7–12.7)	0.70	(0.58, 0.86)		*2282*, *1874*	9.3	(7.9–11.0)	0.71	(0.55–0.91)		*1926*, *2235*
No job (10+ h/week) or not in the last 10 years	11.7	(9.8–14.0)	0.71	(0.55, 0.93)		*1098*, *1045*	7.4	(4.7–11.4)	0.52	(0.31–0.88)		*387*, *426*
Student in full-time education	2.9	(1.8–4.7)	0.18	(0.11, 0.30)		*721*, *443*	2.3	(0.9–5.8)	0.18	(0.07–0.49)		*565*, *456*
Index of multiple deprivation (IMD)—quintile					0.5362						0.2590	
1–2 (least deprived)	13.2	(11.9–14.6)	1.00			*3062*, *2909*	11.1	(9.6–12.8)	1.00			*2197*, *2870*
3	12.5	(10.7–14.5)	0.96	(0.78–1.18)		*1570*, *1371*	8.7	(6.9–11.0)	0.78	(0.57–1.05)		*1080*, *1329*
4–5 (most deprived)	11.7	(10.5–13.1)	0.91	(0.76–1.08)		*3434*, *2772*	9.8	(8.4–11.4)	0.90	(0.71–1.15)		*2276*, *2613*

Denominator: All those who reported having experience of heterosexual sex and who gave a valid answer to the question on experience of infertility.

AOR, adjusted odds ratio; 95% CI, 95% confidence intervals.

^a^Participants aged 21 and older only.

^b^Only those who had a child.

**Figure 1:**
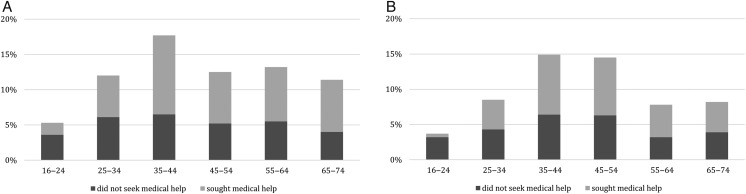
Prevalence of infertility and help seeking, according to age groups. (**A**) Women who had attempted pregnancy with a partner for 12 months or longer. (**B**) Men who had attempted pregnancy with a partner for 12 months or longer.

The experience of infertility was associated with relationship factors. It was highest among those who were married or cohabiting at interview and lowest among those in a non-cohabiting partnership. Among women, a positive association was found between older age at first cohabitation and the experience of infertility. Age-adjusted odds of experiencing infertility were significantly higher among women who first gave birth at age 35 or older (AOR 5.57 (3.97–7.83)) compared with those who did so before age 25. A similar, though slightly weaker, association was observed among men (AOR 3.27 (2.16–4.96)).

We found evidence of associations between infertility and indicators of socio-economic status. Experience of infertility was most common among women with a degree and lowest among those with no academic qualifications while no statistically significant association was observed among men. Prevalence was also higher among those in managerial, professional and technical employment compared with those in routine occupations. No association was found between infertility and area-related deprivation at interview among women or men.

Among women, the only health indicator found to be meaningfully associated with infertility was treatment for depression in the previous 12 months and the association was of only borderline significance (Supplementary data, Table SI). Among men, we found associations with ever having been diagnosed with a sexually transmitted infection (men: AOR 1.39 (1.02–1.88)) and, specifically, with chlamydia (AOR 1.81 (1.15–2.84)).

### Prevalence of help seeking for infertility and associated factors

A total of 57.3% of women and 53.2% of men who had ever experience of infertility had sought medical or professional help as a result (Table II). Figure 1 shows the proportion of women and men with experience of infertility who sought help for the problem by age group at interview.

**Table II:** Prevalence of and factors associated with help seeking among those with experience of infertility by sex.

	Women						Men					
	%	95% CI	AOR	95% CI	*P*-Value	Denominators (unweighted, weighted)	%	95% CI	AOR	95% CI	*P*-Value	Denominators (unweighted, weighted)
All						*** ***						
	**57.3**	**(53.6–61.0)**				***923*, *879***	**53.2**	**(48.1–58.1)**				*470*, *691*
Age group					<0.0001						0.0004	
16–24	32.6	(22.9–44.0)	0.49	0.28–086		*93*, *50*	14.1	(6.8–27.1)	0.17	0.07–0.41		*47*, *36*
25–34	49.5	(43.1–55.9)	1.00			*278*, *157*	49.6	(40.1–59.1)	1.00			*112*, *108*
35–44	63.3	(56.0–70.0)	1.76	1.18–2.63		*208*, *249*	56.9	(47.2–66.2)	1.34	0.78–2.33		*116*, *206*
45–54	58.0	(49.0–66.5)	1.41	0.91–2.20		*136*, *172*	56.1	(45.4–66.3)	1.30	0.73–2.30		*94*, *193*
55–64	58.3	(48.8–67.2)	1.43	0.90–2.27		*120*, *154*	58.1	(43.8–71.1)	1.41	0.71–2.80		*54*, *86*
65–74	64.9	(54.0–74.4)	1.89	1.13–3.15		*88*, *97*	53.1	(38.7–67.0)	1.15	0.57–2.30		*47*, *63*
Relationship history												
Relationship status at interview					<0.0001						0.0049	
Married/cohabiting	60.5	(56.3–64.5)	1.00			*660*, *710*	56.1	(50.5–61.6)	1.00			*348*, *585*
Non-cohabiting partnership	47.5	(35.6–59.7)	0.68	0.40–1.13		*88*, *48*	28.9	(15.2–48.1)	0.35	0.15–0.81		*34*, *30*
No ‘steady’ partner	43.2	(35.1–51.7)	0.43	0.30–0.64		*173*, *120*	39.9	(29.1–51.7)	0.53	0.32–0.89		*85*, *74*
Ever cohabited with a partner					0.0202						0.0017	
Never	26.7	(12.3–48.7)	1.00			*37*, *19*	14.9	(6.1–32.3)	1.00			*25*, *24*
Ever	58.1	(54.3–61.9)	3.10	1.19–8.07		*853*, *832*	54.7	(49.3–59.9)	5.63	1.91–16.58		*424*, *643*
Age at first cohabitation					0.0838						0.0259	
Under 20	51.8	(44.6–59.0)	1.00			*253*, *217*	36.9	(25.3–50.2)	1.00			*68*, *78*
20–29	61.4	(56.7–65.8)	1.44	1.03–2.04		*532*, *533*	56.6	(50.3–62.7)	2.11	1.13–3.93		*298*, *474*
30 or older	53.7	(41.0–66.0)	1.05	0.58–1.91		*68*, *82*	64.0	(49.8–76.1)	2.83	1.26–6.35		*55*, *85*
Reproductive history												
Ever had a child					0.0452						0.3770	
Yes	57.0	(52.7–61.2)	1.00			*683*, *676*	52.3	(46.0–58.6)	1.00			*293*, *471*
No	58.7	(51.5–65.4)	1.14	0.81–1.59		*239*, *203*	54.9	(46.6–62.9)	1.22	0.79–1.88		*177*, *220*
Age at birth of first child^b^					<0.0001						0.0092	
<25	43.0	(36.1–50.1)	1.00			*297*, *245*	32.3	(21.2–45.8)	1.00			*74*, *94*
25–29	59.3	(51.5–66.7)	1.89	1.22–2.92		*184*, *183*	48.9	(37.5–60.3)	1.82	0.86–3.87		*89*, *139*
30–34	65.8	(56.5–74.0)	2.51	1.54–4.09		*129*, *148*	68.0	(54.8–78.8)	4.14	1.85–9.24		*68*, *129*
35 plus	74.6	(62.8–83.7)	3.73	1.99–6.99		*71*, *98*	56.4	(43.0–68.8)	2.42	1.08–5.41		*57*, *109*
Abortion ever					0.954							
No	57.4	(53.4–61.3)	1.00			*788*, *749*						
Yes	56.3	(46.2–66.0)	0.99	0.64–1.52		*131*, *125*						
Socio-economic position												
Educational level^a^					0.0001						0.0158	
Degree	65.5	(58.8–71.7)	1.00			*242*, *255*	66.9	(57.4–75.1)	1.00			*131*, *228*
A-level/equivalent	58.0	(50.1–65.5)	0.73	0.47–1.12		*202*, *197*	47.0	(37.8–56.5)	0.44	0.25–0.77		*131*, *198*
GCSE, O-level or equivalent	55.6	(49.6–61.5)	0.64	0.44–0.94		*360*, *330*	46.4	(37.1–56.0)	0.45	0.26–0.79		*149*, *189*
Foreign or other	7.1	(0.9–38.1)	0.05	0.01–0.41		*11*, *8*	32.8	(9.27–69.9)	0.27	0.05–1.45		*7*, *7.*
None	42.9	(32.9–53.5)	0.33	0.20–0.56		*106*, *89*	45.8	(31.2–61.2)	0.40	0.19–0.86		*52*, *68*
Social class (NS-SEC)					0.0069						0.0136	
Managerial and prof occupations	63.0	(56.9–68.6)	1.00			*312*, *338*	60.2	(52.6–67.4)	1.00			*195*, *322*
Intermediate occupations	62.2	(54.2–69.7)	0.96	0.63–1.46		*201*, *193*	53.8	(41.8–65.4)	0.77	0.42–1.38		*80*, *116*
Semi-routine/routine occupations	47.0	(39.8–54.2)	0.54	0.37–0.81		*256*, *209*	42.8	(34.5–51.6)	0.53	0.33–0.85		*161*, *208*
No job (10+ h/week) or not in the last 10 years	52.3	(42.7–61.8)	0.56	0.35–0.89		*131*, *123*	37.0	(18.7–60.1)	0.34	0.13–0.91		*24*, *32*
Student in full-time education	46.2	(24.0–70.0)	0.64	0.23–1.80		*18*, *13*	78.2	(40.6–94.9)	2.85	0.59–13.90		*8*, *11*
Index of multiple deprivation (IMD)—quintile					0.0583						0.0912	
1–2 (least deprived)	63.3	(57.7–68.6)	1.00			*360*, *383*	60.5	(53.0–67.6)	1.00			*193*, *319*
3	54.6	(46.1–62.8)	0.71	0.47–1.09		*175*, *171*	47.1	(35.2–59.3)	0.60	0.34–1.09		*79*, *116*
4–5 (most deprived)	51.8	(45.7–57.8)	0.67	0.47–0.94		*388*, *326*	46.7	(38.8–54.9)	0.62	0.39–1.00		*198*, *256*

Denominator: all those who reported experience of heterosexual sex and of infertility.

AOR, adjusted odds ratio; 95% CI, 95% confidence intervals.

^a^Participants aged 21 and older only.

^b^Only those who had a child.

Less than one-third (32.6%) of the youngest women (16–24 years) and only 14.1% of the youngest men with experience of infertility reported seeking help. Among both women and men, similar proportions of those aged 35–74 had sought help (women: 58.0–64.9%; men: 53.1–58.1%).

Age-adjusted odds ratios showed help seeking to be higher among those who were currently married or cohabiting compared with those in non-cohabiting or not in ‘steady’ partnerships. Prevalence of seeking help was highest in women who first cohabited in their twenties while, among men, it was highest in those who first cohabited aged 30 or older.

For women, having had a child and older age at first birth were both associated with seeking help for fertility; help seeking was highest among women who became mothers at 35 or older (74.6% (62.8–83.7)). The association between age at first child and help seeking was less marked among men. Of those who had never had a child, 58.7% (95% CI 51.5–65.4) of women and 54.9% (95% CI 46.6–62.9) of men reported having sought help.

Help seeking was significantly associated with indicators of socio-economic position. Women and men with lower levels of education and lower occupational classifications (as indicated by the NS-SEC) were less likely to have sought help. Associations between help seeking and area-level deprivation at interview were of borderline significance among women only.

Health indicators were not associated with likelihood of having sought help for infertility although there was a borderline association suggesting that men who reported regular heavy drinking were slightly less likely to have sought help (Supplementary data, Table SII).

### Associations between experience of infertility and aspects of current well-being and relationship quality among women

Table III presents the associations between experience of infertility and three variables which we have treated as ‘outcomes’ for the purposes of this analysis. We found a positive and significant association between ever experience of infertility and symptoms of depression in the 2 weeks before interview and dissatisfaction with sex life in the past year among women aged 50 and under. These associations remained after controlling for potential confounding factors including age at interview, educational qualification, relationship status at interview, duration of relationship, age at first cohabitation and parental status. We examined whether having children or not moderated the association between experience of infertility and depression and identified no evidence of an interaction (*P* = 0.698). Given the limitations of the data, we do not know whether depression or sexual dissatisfaction was related to the use of fertility treatments. We found no significant association between women's experience of infertility and whether they were happy with their current relationship. Among men, no significant association was observed between the experience of infertility and depression, dissatisfaction with sex life, or happiness with relationship (analysis not shown).

**Table III:** Associations between experience of infertility and aspects of current well-being and relationship quality among women aged 50 and younger at interview.^a^

Women	Depression^b^					Dissatisfied with sex life^c^					Happy in relationship^d^				
	%	AOR	95% CI	*P*-Value	Denominators (unweighted, weighted)	%	AOR	95% CI	*P*-Value	Denominators (unweighted, weighted)	%	AOR	95% CI	*P*-Value	Denominators (unweighted, weighted)
Experience of infertility				0.002					0.031					0.689	
No	11.4	1.00			*5238*, *3964*	13.1	1.00			5230, 3962	62.4	1.00			*3018*, *2561*
Yes	15.3	1.63	1.20–2.21		*667*, *566*	16.2	1.37	1.03–1.83		664, 563	64.2	1.05	0.83–1.34		*482*, *444*

Denominator: all women 50 years and younger with experience of heterosexual sex.

AOR, adjusted odds ratio; 95% CI, 95% confidence intervals.

^a^adjusted for age at interview, highest educational qualification, relationship status at interview, duration of relationship, age at first cohabitation and whether or not a parent.

^b^Positive response to composite question on depressive symptoms in two weeks before interview.

^c^Responded ‘disagree’ or ‘disagree strongly’ with statement: ‘I feel satisfied with my sex life’.

^d^Responded 1 or 2 to question: ‘On a scale of 1–7, how happy or unhappy are you with your relationship with your partner, all things considered?’ (1 = very happy and 7 = very unhappy).

## Discussion

In this study, we provide population prevalence estimates of infertility and help seeking among women and men in Britain using national probability survey data. One in eight women and one in ten men experienced infertility defined by unsuccessfully attempting pregnancy for a year or more. Women and men who settled later with a partner, had higher educational attainment and occupational status and, among those who did have a child, became parents at older ages were more likely to have experienced infertility.

Little more than half of women and men who had experienced infertility had sought medical or professional help for the problem. Those who did so were better educated and in higher status occupations and, if they were parents, were more likely to have had children at older ages. These characteristics were more marked among women than men. Women aged 50 or younger who had experience of infertility were more likely to report recent symptoms of depression and dissatisfaction with their sex life. We found that ever experience of infertility and of help seeking were associated with few current health factors for women or men.

A strength of this study is the size of the sample and the fact that it is population-based. Natsal-3 includes data on sexual and reproductive health as well as behavioural and relationship variables less common in health surveys. A limitation is that, although we measure ever experience of infertility, some explanatory variables refer to recent time frames. As data are cross-sectional, it is not possible to establish temporality or infer causality and we cannot establish the age of participants when they experienced infertility or sought help, or the timing of these experiences relative to having children for those who did. Self-reported data may be subject to recall bias. It was not possible to explore associations between infertility and participant ethnicity as numbers were too small.

Our estimates of infertility are broadly in line with those found by previous studies although prevalence estimates differ as a result of diverse definitions and study populations. Earlier estimates of women experiencing infertility range from around 1 in 5 (Bhattacharya *et al*., 2009; Cabrera-León *et al*., 2015) to around 1 in 10 (Evers, 2002) with the National Women's Health Study reporting about 1 in 6 (Oakley *et al*., 2008). Estimates of couples' experience of infertility also vary. Hull *et al*. (1985) estimated it to be around one in six while NICE cites estimates of one in seven (National Institute for Health and Clinical Excellence, 2013).

A large body of literature describes the trend among women in developed countries to delay having children (Schmidt *et al*., 2012) and it is proposed that this changing fertility tempo is becoming a global phenomenon (Rosero-Bixby *et al*., 2009; Sobotka, 2010).

Our finding that infertility was more commonly experienced by married or cohabiting participants probably reflects the fact that those in stable relationships are more likely to have attempted pregnancy and therefore become aware of fertility problems. The much lower prevalence among young people reflects in part that they may never have tried to get pregnant. Previous studies have noted the extended period of transition to adulthood (Stone *et al*., 2014) and the widening intervals between the key reproductive events of first sex, first cohabitation and first birth (Wellings *et al*., 2013). Researchers taking a life course perspective (Morgan and Rackin, 2010; Berrington and Pattaro, 2014) have observed discrepancies between individuals' fertility intentions and their subsequent family size, highlighting the complex interaction of childhood socialization with personal and structural factors. Influences on postponement for women include the increase in women's participation in further education (Andersson *et al*., 2008; Ní Bhrolcháin and Beaujouan, 2012) and in career development (Martin, 2000; Morris *et al.,* 2011), the absence of a ‘suitable’ partner (Proudfoot *et al*., 2009), partner's expectations (Iacovou and Tavares, 2011) and perceptions of how parenthood will reduce individual autonomy (Liefbroer, 2005).

Our estimate that 57% of women sought medical help for infertility is close to that reported by an international review which estimated that 56% of women in more developed countries sought help (Boivin *et al*., 2007), and by a Finnish study (Terävä *et al*., 2008) which found that 57% of all subfertile women did so. Other studies (Greil and McQuillan, 2004; Morris *et al*., 2011; Chandra *et al*., 2014) have reported higher and lower estimates but, because of differences in study groups and outcome measures, comparison is not possible.

Our analyses show that women aged 50 or under who experienced infertility were more likely to report recent symptoms of depression and dissatisfaction with their sex life. Infertility is associated with psychological distress (Cousineau and Domar, 2007; Greil *et al.,* 2011) and patients find the process of undergoing infertility treatment and its uncertain outcome stressful (Sbaragli *et al*., 2008; Volgsten *et al*., 2008; Schmidt *et al*., 2013). The longer term impact of infertility on mental health and sexual well-being is less well known. A Danish study shows a relationship between unsuccessful ART and severe depressive symptoms a year after initiating treatment (Lund *et al*., 2009) and another, also Danish, found women who did not have a child after fertility treatment were more likely to commit suicide than those who did (Kjaer *et al*., 2011). Previous research has found associations between undergoing treatment for infertility and sexual dissatisfaction, particularly among women (Millheiser *et al*., 2012; Wischmann, 2010; Marci *et al*., 2012).

The large minority of research participants who experienced infertility but did not seek medical help is of concern, as are the marked inequalities in help seeking between those who are well qualified and in high status employment and those who are not. These findings are in line with other studies from Europe, North America and Australia (Terävä *et al*., 2008; Bushnik *et al*., 2012; Chambers *et al*., 2013; Chandra *et al*., 2014). Several explanations for not seeking (or pursuing) help for infertility have been suggested, including not understanding or acknowledging that a problem exists (White *et al*., 2006), fear of being labelled infertile (Bunting and Boivin, 2007), concerns about the cost of treatment (Eisenberg *et al*., 2010), lack of intent to conceive (Greil and McQuillan, 2004) and the physical and psychological burden of treatment (Verberg *et al*., 2008). These do not provide a clear rationale for why there should be a distinction between indicators of social status and the likelihood of seeking help, although it has been suggested that acknowledging lack of conception as a problem to be solved is a motivation for seeking treatment and that highly educated women may be better informed about how long conception might typically take (Morris *et al*., 2011).

Interventions to encourage help seeking include raising public awareness about reproductive risks and strategies to minimise them (Macaluso *et al*., 2010), general practitioners taking opportunities to discuss fertility with patients (Davies, 2015), greater access to fertility treatments (Bunting and Boivin, 2007) and an acknowledgement of the psychosocial impacts of infertility, including the long-term effects, by health practitioners and the availability of appropriate support (Hinton *et al*., 2012).

## Supplementary data

Supplementary data are available at http://humrep.oxfordjournals.org/.

## Authors' roles

J.D., K.W., C.T. and M.P. conceived the paper; all authors contributed to data acquisition and interpretation; M.P., L.J.G. and K.G.J. conducted the statistical analysis; J.D. wrote the first draft with contributions from all other authors. All authors approved the final draft.

## Funding

The study was funded by grants from the Medical Research Council and the Wellcome Trust with contributions from the Economic and Social Research Council and Department of Health. K.G.J. was supported by a National Institute for Health Research Methods Fellowship. Funding to pay the Open Access publication charges for this article was provided by MRC and Wellcome Trust.

## Conflict of interest

AMJ is a Governor of the Wellcome Trust. Other authors declare no conflicts of interest.

## Supplementary Material

Supplementary Data
