# Supplementary material for: Prevalence of infertility and help seeking among 15 000 women and men
Source: Hum Reprod. 2016 Aug 19;31(9):2108–18. doi: 10.1093/humrep/dew123 (PMC4991655; doi:10.1093/humrep/dew123)
Supplement: Supplementary Data [file supp_31_9_2108__index.html]

Prevalence of infertility and help seeking among 15 000 women and men — Prevalence of infertility and help seeking among 15 000 women and men — Supplementary Data 

# Prevalence of infertility and help seeking among 15 000 women and men

## Supplementary Data

Supplementary Data

- Supplementary Table 1 - pdf file
- Supplementary Table 2 - pdf file
