# Supplementary material for: Prevalence of infertility and help seeking among 15 000 women and men
Source: Hum Reprod. 2016 Aug 19;31(9):2108–18. doi: 10.1093/humrep/dew123 (PMC4991655; doi:10.1093/humrep/dew123)
Supplement: Supplementary Data [file supp_dew123_dew123supp_table1.pdf]

Human Reproduction, Vol.0, No.0 , 2016
doi:10.1093/humrep/dew123
human reproduction
SUPPLEMENTARY DATA

Supplementary Table SI Prevalence of health-related factors associated with infertility by sex.

	Women						Men					
	Pregnancy attempt 12 months or longer		Age-adjusted regression				Pregnancy attempt 12 months or longer		Age-adjusted regression			
	%	95% CI	AOR	95% CI	*P*-Value	Denominators (unweighted, weighted)	%	95% CI	AOR	95% CI	*P*-Value	Denominators (unweighted, weighted)
All												
	**12.5%**	**(11.7–13.3)**				*8066, 7052*	**10.1%**	**(9.2–11.1)**				*5553, 6811*
Health status and behaviour												
Longstanding illness, disability or infirmity at interview					0.5323						0.7067	
None	11.9	(10.9–12.9)	1.00			*5528, 4654*	9.6	(8.4–10.8)	1.00			*3904, 4625*
Non-limiting	13.6	(11.4–16.2)	1.10	(0.88–1.38)		*1109, 1046*	11.6	(9.2–14.5)	1.14	(0.83–1.57)		*754, 1050*
Limiting	13.6	(11.7–15.9)	1.10	(0.89–1.35)		*1428, 1351*	11.0	(8.8–13.8)	1.07	(0.79–1.46)		*893, 1135*
Body mass index at interview					0.1552						0.7047	
Underweight/normal)	11.5	(10.3–12.7)	1.00	-		*4078, 3378*	9.4	(8.1–11.0)	1.00	-		*2480, 2709*
Overweight	13.4	(11.8–15.1)	1.14	(0.95–1.38)		*2041, 1913*	10.2	(8.7–11.8)	1.03	(0.80–1.32)		*1943, 2612*
Obese	14.1	(12.1–16.4)	1.22	(0.98–1.51)		*1443, 1347*	11.3	(9.2–13.8)	1.13	(0.85–1.50)		*937, 1290*
Treated for depression in the last 12 months					0.0780						0.1560	
No	12.2	(11.3–13.1)	1.00			*6938, 6141*	10.0	(9.0–11.0)	1.00			*5164, 6390*
Yes	14.4	(12.0–17.2)	1.23	(0.98–1.54)		*1126, 909*	12.9	9.2–17.6)	1.32	(0.90–1.95)		*387, 420*
Ever smoked regularly					0.7471						0.1897	
No	12.3	(11.2–13.5)	1.00	-		*3993, 3648*	10.7	(9.3–12.2)	1.00	-		*2535, 3120*
Yes	12.6	(11.5–13.9)	1.03	(0.88–1.20)		*4073, 3404*	9.7	(8.5–11.0)	0.87	(0.70–1.07)		*3018, 3691*
Binge drinking[a]					0.2716						0.9991	
Never/rarely	13.1	(12.1–14.2)	1.00	-		*5121, 4682*	10.3	(9.1–11.6)	1.00	-		*3011, 3924*
Monthly	11.5	(9.4–13.8)	0.93	(0.73–1.19)		*1121, 846*	9.6	(7.5–12.2)	0.99	(0.72–1.36)		*960, 1074*
Minimum weekly	10.3	(8.0–13.1)	0.80	(0.60–1.06)		*942, 772*	9.8	(7.9–12.1)	1.00	(0.75–1.32)		*1222, 1384*
Ever diagnosed with an STI					0.2631						0.0352	
No	12.3	(11.5–13.3)	1.00	-		*6650, 5982*	9.7	(8.7–10.8)	1.00	-		*4780, 5870*
Yes	13.1	(11.1–15.5)	1.13	(0.91–1.40)		*1363, 1023*	12.8	(10.0–16.3)	1.39	(1.02–1.88)		*724, 879*
Ever diagnosed with chlamydia					0.8825						0.0102	
No	12.5	(11.6–13.4)	1.00	-		*7357, 6585*	9.9	(9.0–11.0)	1.00	-		*5189, 6442*
Yes	11.2	(8.5–14.5)	0.98	(0.71–1.34)		*612, 379*	15.0	(10.2–21.4)	1.81	(1.15–2.84)		*275, 257*

AOR, adjusted odds ratio; 95% CI, 95% confidence intervals.
[a]Binge drinking defined as ‘how often do you drink >6 (for women) or >8 (for men) units on one occasion?’

Supplementary Table SII **Prevalence of health-related factors associated with help seeking for infertility by sex.**

	Women						Men					
	%	95% CI	AOR	95% CI	*P*-Value	Denominators (unweighted, weighted)	%	95% CI	AOR	95% CI	*P*-Value	Denominators (unweighted, weighted)
All												
	**57.3**	**(53.6–61.0)**				***923, 879***	**53.2**	**(48.1–58.1)**				*470, 691*
Health status and behaviour												
Longstanding illness, disability or infirmity					0.2087						0.7814	
None	58.1	(53.7–62.5)	1.00			*600, 553*	52.3	(46.0–58.4)	1.00			*305, 443*
Non-limiting	51.9	(42.6–61.0)	0.68	0.44–1.04		*136, 142*	57.5	(45.2–68.9)	1.11	0.63–1.96		*79, 122*
Limiting	59.1	(50.8–66.9)	0.91	0.62–1.35		*187, 184*	51.8	40.0–63.4)	0.87	0.50–1.52		*85, 125*
Body mass index at interview					0.4549						0.7502	
Underweight	61.9	(39.3–80.3)	1.29	0.52–3.18		*22, 17*	44.3	(10.9–83.7)	0.77	0.12–4.86		*6, 9*
Normal	57.5	(52.1–62.7)	1.00			*401, 370*	54.9	(46.5–63.1)	1.00			*175, 247*
Overweight	61.2	(54.3–67.6)	1.12	0.78–1.60		*261, 256*	50.2	(42.0–58.3)	0.77	0.47–1.25		*175, 266*
Obese	52.8	(44.5–60.9)	0.80	0.54–1.19		*191, 190*	54.6	(43.6–65.1)	0.93	0.53–1.64		*98, 145*
Treated for depression in the last 12 months					0.6310						0.4820	
No	57.8	(53.8–61.7)	1.00			771, 748	53.7	(48.4–58.9)	1.00			426, 637
Yes	54.8	(45.8–63.6)	0.91	0.61–1.35		152, 131	46.6	(30.1–63.9)	0.77	0.37–1.60		44, 54
Ever smoked regularly					0.2400						0.1310	
No	59.6	(54.4–64.7)	1.00			*438, 448*	57.3	(50.0–64.3)	1.00			*209, 334*
Yes	54.9	(49.6–60.1)	0.84	0.62–1.13		*485, 431*	49.3	(42.4–56.2)	0.73	0.48–1.10		*261, 357*
Binge drinking[a]					0.1592						0.0532	
Never or less than monthly	56.1	(52.0–60.1)	1.00			*726, 702*	58.1	(51.7–64.3)	1.00			*289, 452*
Monthly	59.6	(49.0–69.3)	1.28	0.81–2.01		*112, 97*	42.4	(30.9–54.8)	0.56	0.32–0.99		*78, 103*
At least weekly	65.9	(53.9–76.1)	1.57	0.93–2.63		*84, 79*	44.7	(34.1–55.9)	0.61	0.36–1.02		*103, 136*
Ever diagnosed with an STI					0.5740						0.2220	
No	57.3	(53.2–61.3)	1.00			*755, 738*	55.1	(49.6–60.5)	1.00			*385, 569*
Yes	58.1	(49.3–66.5)	1.12	0.75–1.67		*162, 134*	45.8	(33.9–58.2)	0.71	0.42–1.23		*79, 113*
Ever diagnosed with chlamydia					0.5220						0.1180	
No	57.9	(54.0–61.71	1.00			*841, 822*	55.0	(49.7–60.1)	1.00			*429, 640*
Yes	49.2	(36.0–62.6)	0.82	0.45–1.49		*68, 42*	34.9	(18.4–56.1)	0.49	0.20–1.20		*33, 39*

AOR, adjusted odds ratio; 95% CI, 95% confidence intervals.

[a]Binge drinking defined as 'how often do you drink >6 (for women) or >8 (for men) units on one occasion?'
